# Tumor-Derived Extracellular Vesicles Induce CCL18 Production by Mast Cells: A Possible Link to Angiogenesis

**DOI:** 10.3390/cells11030353

**Published:** 2022-01-21

**Authors:** Irit Shefler, Pazit Salamon, Tali Zitman-Gal, Yoseph A. Mekori

**Affiliations:** 1 The Herbert Mast Cell Disorders Center, Laboratory of Allergy and Clinical Immunology, Meir Medical Center, Kfar Saba 4428164, Israel; pazit.salamon@clalit.org.il (P.S.); ymekori@gmail.com (Y.A.M.); 2 Sackler School of Medicine, Tel Aviv University, Tel Aviv 6997801, Israel; tali.gal@clalit.org.il; 3 Department of Nephrology and Hypertension, Meir Medical Center, Kfar Saba 4428164, Israel; 4 Tel Hai College, Tel Hai 1220800, Israel

**Keywords:** mast cell, lung tumor, extracellular vesicles, CCL18, angiogenesis

## Abstract

Mast cells (MCs) function as a component of the tumor microenvironment (TME) and have both pro- and anti-tumorigenic roles depending on the tumor type and its developmental stage. Several reports indicate the involvement of MCs in angiogenesis in the TME by releasing angiogenic mediators. Tumor cells and other cells in the TME may interact by releasing extracellular vesicles (EVs) that affect the cells in the region. We have previously shown that tumor-derived microvesicles (TMVs) from non-small-cell lung cancer (NSCLC) cells interact with human MCs and activate them to release several cytokines and chemokines. In the present study, we characterized the MC expression of other mediators after exposure to TMVs derived from NSCLC. Whole-genome expression profiling disclosed the production of several chemokines, including CC chemokine ligand 18 (CCL18). This chemokine is expressed in various types of cancer, and was found to be associated with extensive angiogenesis, both in vitro and in vivo. We now show that CCL18 secreted from MCs activated by NSCLC-TMVs increased the migration of human umbilical cord endothelial cells (HUVECs), tube formation and endothelial- to-mesenchymal transition (EndMT), thus promoting angiogenesis. Our findings support the conclusion that TMVs have the potential to influence MC activity and may affect angiogenesis in the TME.

## 1. Introduction

Mast cells (MCs) are known as pivotal effector cells in allergic responses. However, emerging data indicate their important role in establishing innate and adaptive immune responses [1]. MCs are also present in the microenvironment of various tumors and function as a component of the tumor microenvironment (TME) [2,3]. Within the TME, MCs have both pro- and anti-tumorigenic roles depending on the tumor type and its developmental stage [4,5].

By releasing different mediators, MCs can promote tumor growth by affecting angiogenesis and tissue remodeling, and by modulating the host immune response. The anti-tumorigenic effects of MCs include direct growth inhibition, immunologic stimulation and decreased cell mobility [4,6].

Angiogenesis, the formation of new blood vessels from pre-existing ones, facilitates tumor progression, growth and aggressiveness [7,8]. Increasing evidence is identifying the contribution of MCs in the TME to angiogenesis. This can be accomplished by MCs releasing angiogenic mediators following activation via several soluble factors present in the TME. These angiogenic mediators include vascular endothelial growth factor (VEGF), basic fibroblast growth factor (FGF), IL-8 and tumor necrosis factor-α (TNF-α) [6,9].

One way tumor cells and other cells in the tumor microenvironment interact may be by releasing extracellular vesicles (EVs) that affect the cells in the region [10,11]. Indeed, we have previously reported that tumor-derived microvesicles (TMVs) from non-small-cell lung cancer (NSCLC) cells can activate MCs to release several mediators, including TNF-α and monocyte chemoattractant protein 1 (MCP-1)/ CC chemokine ligand 2 (CCL2), as well as enhancing both their chemotactic and chemokinetic activity [12].

In the present study, we characterized the MC expression of other mediators after exposure to TMVs derived from NSCLC. Whole-genome expression profiling disclosed the production of several chemokines. One of which, CC chemokine ligand 18 (CCL18), known to be released by tumor-associated macrophages (TAMs), was found to be associated with extensive angiogenesis, both in vitro and in vivo in breast cancer [13]. Along this line, we found that CCL18 secreted from MCs activated by NSCLC-TMVs increased HUVEC migration, tube formation and EndMT, thus promoting angiogenesis. Therefore, our findings support the conclusion that EVs derived from tumor cells have the potential to influence the activity of MCs and may affect angiogenesis in the TME.

## 2. Materials and Methods

### 2.1. Antibodies and Reagents

The following antibodies were used; N-cadherin (Cell Signaling Technology, Danvers, MA, USA), anti-tubulin (Sigma-Aldrich, St. Louis, MO, USA), 10 μg/mL anti-CCL18 (Ori Gene Technology, Rockville, MD, USA), 10 μg/mL monoclonal mouse anti-rabbit Ig (Dako, Glostrup, Denmark), HRP-conjugated secondary antibodies (Jackson ImmunoResearch Laboratories, West Grove, PA, USA) and recombinant CCL18 (PeproTech Asia, Rehovot, Israel).

### 2.2. Cell Culture

Cell culture reagents were purchased from Biological Industries (Beit Haemek, Israel). Human LAD2 MCs, kindly provided by Dr. A.S. Kirshenbaum [14], were maintained in StemPro-34^®^ SFM (GIBCO^TM^ Invitrogen Corporation, Grand Island, NY, USA) supplemented with 2 mM L-glutamine, 50 μg/mL streptomycin, 100 U/mL penicillin, 12.5 U/mL nystatin and 100 ng/mL recombinant human stem cell factor (PeproTech Asia, Rehovot, Israel) as previously described [15,16]. Human adenocarcinoma cell line A549, kindly provided from the Lung Cancer Research Laboratory, Meir Medical Center, Kfar Saba, Israel, was cultured in Dulbecco’s modified Eagle medium (DMEM) supplemented with 10% heat-inactivated FBS, 2 mM L-glutamine, 100 U/mL penicillin, 100 μg/mL streptomycin and 12.5 U/mL nystatin.

Endothelial cells were isolated from human umbilical cords (HUVECs) obtained from the Obstetrics Unit at Meir Medical Center, Kfar Saba, Israel, as described previously [17]. The study was approved by the Meir Medical Center Ethics Committee (approval no. 0074-11-MMC) and parturient women provided written informed consent. Briefly, fresh umbilical cord veins were rinsed with PBS, filled with a solution of collagenase H (0.3%; Merck, Darmstadt, Germany) in PBS, and incubated at 37 °C for 10 min. The collagenase solutions were removed and the veins washed with PBS. HUVECs were collected by centrifugation at 1200 rpm for 10 min and then seeded in culture flasks. The HUVECs were grown in M-199 medium supplemented with 20% FCS, 100 U/mL penicillin, 100 μg/mL streptomycin and 0.25 µg/ml amphothericin (Biological Industries)), 5 U/mL heparin and 25 µg/mL endothelial mitogen ((Merck) under standard cell culture conditions (humidified atmosphere, 5% CO_2_, 37 °C). Confluent cultures of HUVECs at passage 2–4 were used for the experiments.

### 2.3. Isolation of Tumor-Derived Microvesicles (TMVs)

TMVs were isolated from conditioned media collected from 80% confluent cultures of the NSCLC A549 cell line as described previously [15]. Briefly, supernatants from cells were centrifuged at 800× *g* for 5 min and then centrifuged at 4500× *g* for 5 min to discard large debris. TMVs were isolated after centrifugation at 20,000× *g* for 60 min at 4 °C, followed by washing and resuspension in PBS. Ultrastructural analysis of the isolated MV demonstrated membrane-coated round vesicles ranging in size from 100 to 800 nm in diameter, that exposed phosphatidylserine on their surface [15]. The TMV concentration was measured at 280 nm using a NanoDrop spectrophotometer (Thermo Fisher Scientific Waltham, MA, USA; NanoDrop™ 1000). The quantity of protein in TMVs was similar in all analyzed samples.

### 2.4. Mast Cell Activation

LAD2 MCs were activated by incubating with 100 μg/mL TMVs isolated from the A549 cell line for 48–72 h.

### 2.5. RNA Isolation

Total RNA was extracted from LAD2 MCs activated with 100 μg/mL TMVs isolated from the A549 cell line for 24–48 h, using Direct-Zol^TM^ RNA MiniPrep Plus kit (Zymo research, Irvine, CA, USA), according to the manufacturer’s protocol.

### 2.6. High-Throughput Sequencing and Data Analysis

RNA concentration, purified from untreated LAD2 cells or LAD2 cells treated with A549-TMVs for 48 h (in triplicate), was measured using a Qubit 4 Fluorometer (ThermoFisher Scientific). RNA quality was measured using Agilent 2200 TapeStation (Agilent, Santa Clara, CA, USA). RNA sequencing libraries were prepared using the CEL-Seq2 protocol, as published by Hashimshony et al. [18] with minor modifications. Instead of single cells as input, 2 ng purified RNA was used as input for library preparation. The CEL-Seq2 libraries were sequenced on an Illumina HiSeq 2500 sequencer (Illumina, San Diego, CA, USA). RNA measurements, library preparation and sequencing were performed by the Technion Genome Center, Technion, Israel. Normalization and differential expression analysis were performed by DESeq2 R package, version 1.18.1. Data were normalized and summarized with the Wald test provided by DESeq2.

Genes of interest that were differentially expressed (Benjamini–Hochberg adjusted *p*-values < 0.05 and fold difference cutoff 1.5) were retrieved. For gene ontology (GO) enrichment, the analysis was assessed using three different Bioinformatics tools: ToppGene (www.toppgene.cchmc.org, accessed on 30 March 2020), GOrilla (www.cbl-gorilla.cs.technion.ac.il, accessed on 30 March 2020) and string (www.string-db.org, accessed on 30 March 2020).

### 2.7. Real-Time PCR

cDNA was synthesized using the High-Capacity cDNA Reverse Transcription Kit (Applied Biosystems, Waltham, MA, USA). Gene expression was determined with Fast Real-Time PCR using an ABI 7500 Thermal Cycler (Applied Biosystems). Expression of *CCL18* and *CCL4* genes was measured using specific TaqMan probes (Applied Biosystems; Hs00268113_m1 and Hs99999148_m1, respectively). Expression of β-glucuronidase gene (*GUSB*) was used as a housekeeping gene for analysis of changes in the cycle threshold values.

### 2.8. Human Cytokine Assay

Supernatants, obtained from the different culture conditions of MCs activated as described in Section 2.4, were examined for the release of CCL18 using a commercial ELISA kit (DuoSet; R&D systems, Minneapolis, MN, USA) and for CCL4 with a commercial ELISA kit (Development kit, PeproTech Asia).

### 2.9. SDS-PAGE and Immunoblotting

Cellular extracts were separated with SDS-PAGE using 10% polyacrylamide gels and processed for immunoblotting as described in detail elsewhere [19]. Immunoreactive bands were visualized using the LAS-3000 imaging system (Fujifilm Corp., Tokyo, Japan).

### 2.10. Cell Proliferation

Proliferation was assessed on HUVECs using cell proliferation reagent WST-1 (Roche, Basel, Switzerland), according to the manufacturer’s protocol.

### 2.11. Wound Healing Assay

HUVECs were seeded onto 96-well plates at a concentration of 20,000 cells/well. After 24 h, cells were wounded by scratching with a sterile pipette tip lengthwise through the well. The cells were washed with PBS and incubated with supernatants obtained from the different culture conditions described in Section 2.7. Wound closure was monitored by microscopy immediately after cells were scratched (time 0) and at 4 h after wounding. Results are presented as the relative percentage of closure.

### 2.12. In Vitro Angiogenesis Assay by Tube Formation

Matrigel tube-formation assay was performed in vitro on HUVECs. Briefly, Matrigel (BD, NJ, USA) was thawed on ice, plated into 96-well culture plates and allowed to polymerize for 1 h at 37 °C. HUVECs (1 × 10^4^ cells in 150 μL) pretreated with supernatants obtained from the different culture conditions described in Section 2.4 were seeded on the polymerized Matrigel-coated surface. After 18 h of incubation at 37 °C, the HUVECs aligned to form the cords that ultimately became the pattern for new capillary structures. The branch points of the tube structures were counted using image J (https://imagej.nih.gov, accessed on 10 January 2021).

### 2.13. Statistical Analysis

Results are presented as mean ± SE. Unpaired Student’s *t*-tests were used to analyze the data. A *p*-value less than 0.05 was considered statistically significant.

## 3. Results

### 3.1. TMVs Derived from Lung Cancer Cells Induced CCL18 Release from Mast Cells

TMVs derived from NSCLC cells were previously shown to internalize into MCs and stimulate them to release cytokines such as TNF-α and MCP-1/CCL2, as well as enhance their chemotactic and chemokinetic activity [12]. In order to further characterize the impact of NSCLC-derived TMVs on MC function, we examined the global gene expression profiles induced by incubating LAD2 cells with TMVs derived from A549 cells, a known NSCLC cell line.

Following the stimulation of LAD2 cells with these TMVs, microarray analysis disclosed a significant upregulation of 330 genes and a downregulation of 662 genes (fold-change cutoff 1.5; *p* < 0.05; Supplementary Tables S1 and S2, respectively). To identify the pathways that underlie the biological processes, we used several bioinformatics tools. The gene expression profile of A549-TMV-activated LAD2 cells was compared with that of untreated LAD2 cells. The most relevant annotations are presented in Table 1. The genes upregulated in the cluster of “cellular response stimulus” are depicted in Table 2. Of note, Table 2 discloses a 1.89-fold increase in TNF expression, which is in line with our previous results on the expression of this cytokine by A549-TMVs [12].

**Figure 1:**
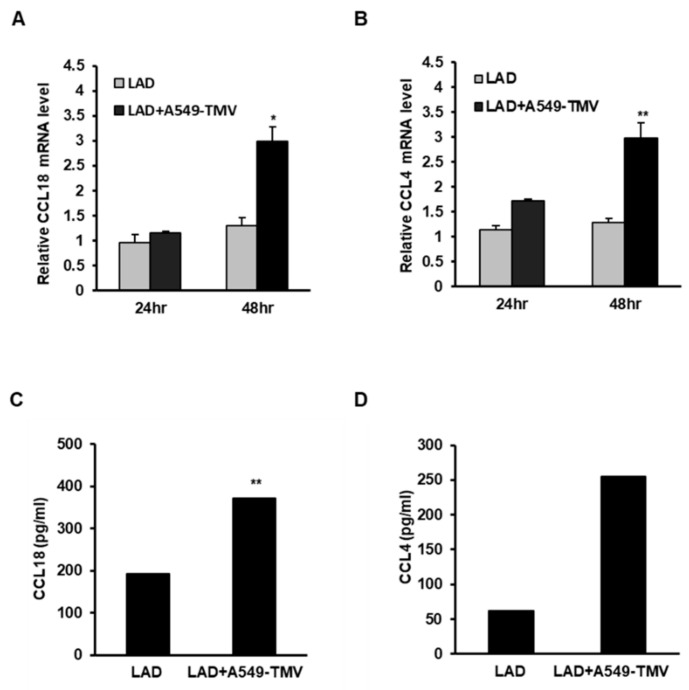
Validation of the effect of NSCLC-TMVs on selected gene expression in LAD2 cells. (**A**,**B**) LAD2 cells were stimulated with 100 μg/mL A549-TMVs for 24–48 h for RNA analysis and for 72 h for protein analysis. mRNA expression of *CCL18* and *CCL4* was assayed using real-time PCR and normalized to *GUSB*. (**C**,**D**) Protein release was assayed by ELISA. Data are presented as means ± SEs of three independent experiments done in duplicate (* *p* < 0.05; ** *p* < 0.01).

Microarray data were validated by analyzing the expression of the chemokines *CCL18* and CC chemokine ligand 4 (*CCL4*), which are presented in Table 2, following the incubation of LAD2 cells with A549-TMVs using real-time quantitative PCR (Figure 1A,B) and ELISA (Figure 1C,D). As shown in Figure 1, the RNA levels of *CCL18* and *CCL4* were higher after 48 h incubation (Figure 1A,B), while cytokine release was detected after 72 h (Figure 1C,D), confirming the microarray data for these genes.

### 3.2. CCL18 Derived from TMV-Stimulated Mast Cells Induced HUVEC Migration

Angiogenesis is a phenomenon that includes several different processes, such as endothelial cell proliferation, differentiation and migration, which lead to the formation of new blood vessels [20].

**Figure 2:**
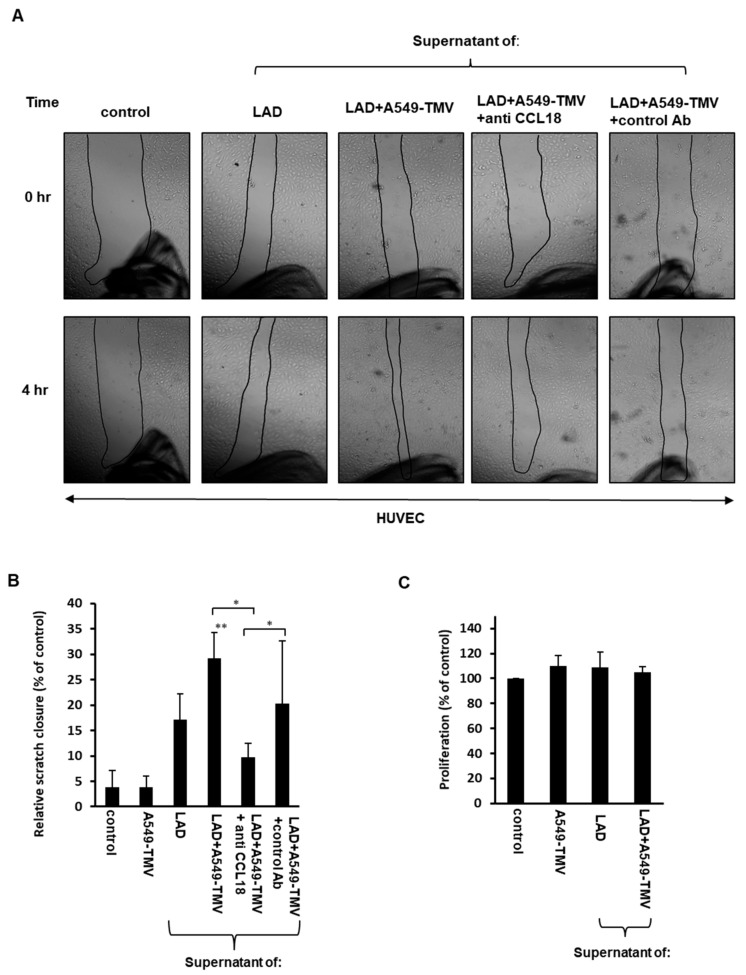
CCL18 released from NSCLC-TMV-stimulated mast cells induced HUVEC migration but not proliferation. (**A**) The effect of supernatants obtained from LAD2 cells activated by A549-TMVs on HUVEC migration was assessed by scratch assay. Scratch assay closure was photographed immediately (0 h) and after 4 h (magnification × 40). (**B**) Results are presented as percent of control in bar graphs. (**C**) The effect of supernatants obtained from LAD2 cells activated by A549-TMVs on HUVEC proliferation. Results are presented as percent of control. Data are presented as means ± SEs of three independent experiments done in duplicates (* *p* < 0.05; ** *p* < 0.01).

Recent studies have demonstrated that the chemokine CCL18 is able to promote endothelial cell migration [13,21]. Therefore, we examined whether CCL18 released by MCs activated by A549-derived TMVs is able to promote endothelial cell migration. Thus, we assayed the migration capabilities of the HUVECs treated with supernatants of A459-TMV-activated MCs as compared to supernatants from naïve MCs, using a scratch assay. Analysis of the assay revealed a significant acceleration in the scratch closure (about 50%) of HUVECs incubated for 4 h with supernatants derived from activated MCs, in comparison to HUVECs incubated with supernatants from naïve MCs (Figure 2A,B).

To further investigate whether this effect is due to CCL18 present in the supernatants of MCs activated by A549-TMVs, we added neutralizing anti-CCL18 mAb to these supernatants. Indeed, addition of the neutralizing mAb led to significant inhibition of the HUVEC scratch closure (65%), suggesting the involvement of CCL18 in HUVEC migration (Figure 2A,B).

Additionally, we evaluated whether supernatants of A549-TMV-activated MCs may induce HUVEC proliferation. Incubation of HUVECs with these supernatants did not affect their proliferation, as compared to HUVECs incubated with supernatants obtained from naïve MCs (Figure 2C).

These results indicate that CCL18 released from MCs activated by A459-TMVs is involved in greater HUVEC migration but not proliferation.

### 3.3. CCL18 Derived from TMV-Stimulated Mast Cells Induced HUVEC Tube Formation

**Figure 3:**
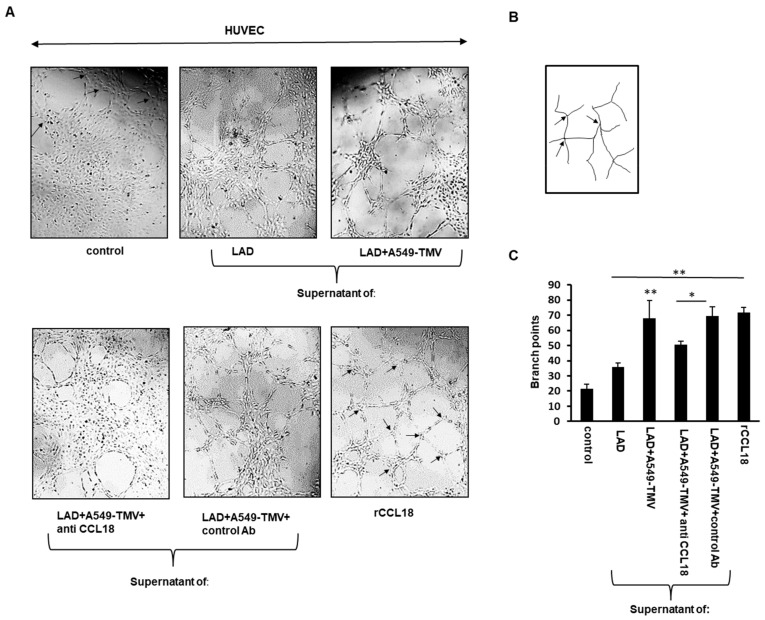
CCL18 released from NSCLC-TMV-stimulated mast cells promoted tube formation. (**A**) Representative images of Matrigel tube-formation assay of HUVECs treated as indicated. The arrows in “control” and “rCCL18” depict branch points. (**B**) Illustration of the image-processing procedure for analyzing the number of branch points. (**C**) Quantitative analysis of tube formation was performed by measuring the branch points of tubular structures formed. Data are presented as means ± SEs of three independent experiments done in duplicate (* *p* < 0.05; ** *p* < 0.01).

The effect of CCL18 released by A459-TMV-activated MCs on angiogenesis was also examined by the ability of HUVECs to form three-dimensional capillary-like structures on Matrigel. As shown in Figure 3, the endothelial tubular structures formed by HUVECs treated with supernatants of A459-TMV-activated MCs exhibited three times more branch points compared to HUVECs treated with media alone and twice as many branch points as HUVECs treated with supernatants of naïve MCs. Treatment of HUVECs with CCL18 served as positive control (Figure 3). Addition of neutralizing anti-CCL18 mAb to the supernatants of A459-TMV-activated MCs resulted in a significant, albeit incomplete, inhibition (approximately 30%) in the formation of branch points, suggesting the involvement of CCL18 and additional mediators in this process.

These results indicate that CCL18 released from MCs activated by A459-TMVs is responsible, at least in part, for the formation of more HUVEC tubular structures and angiogenesis.

### 3.4. CCL18 Derived from TMV-Stimulated MCs Enhanced Endothelial–Mesenchymal Transition in HUVECs

Epithelial-to-mesenchymal transition (EMT) is usually a crucial step in the early stage of cancer metastasis, characterized by downregulation of the epithelial marker E-cadherin and upregulation of the mesenchymal marker N-cadherin [22]. At the angiogenic front, endothelial cells might undergo EMT, which is termed endothelial-to–mesenchymal transition (EndMT) and promotes angiogenic sprouting [21,23].

**Figure 4:**
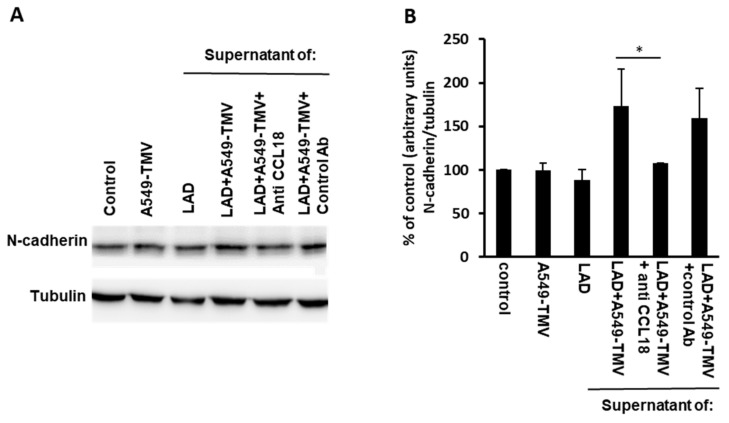
CCL18 released from NSCLC-TMV-stimulated mast cells enhanced EndMT in HUVECs. (**A**) HUVECs were incubated for 24 h as indicated. N-cadherin levels were analyzed by immunoblotting. (**B**) Densitometry analysis of N-cadherin. These results are representative of three independent experiments. Data are presented as mean ± SE (* *p* < 0.05).

Therefore, we analyzed the expression levels of N-cadherin in HUVECs that were incubated for 24 h with supernatants obtained from activated MCs. As can be seen in Figure 4, N-cadherin expression increased when HUVECs were treated with supernatants obtained from activated MCs as compared to supernatants from naïve MCs. To evaluate the involvement of CCL18 released in A549-TMV-activated MC supernatants, a neutralizing anti-CCL18 mAb was added to these samples, resulting in a 40% decrease in N-cadherin expression in HUVECs.

Taken together, these results suggest that CCL18 secreted from A549-TMV-activated MCs induce, in part, EndMT in HUVECs, to produce functional changes consistent with angiogenesis.

## 4. Discussion

Accumulated data reveal the presence of MCs in the TME. Increased MC density in the TME has been associated with both good and poor prognoses, depending on the tumor type and stage [4,5]. MCs play a multifaceted role in the TME by modulating various events in tumor progression, including proliferation and survival, angiogenesis, invasiveness and metastasis [6].

In the TME, MCs can be activated in response to several soluble factors that can drive MC recruitment and activation [6]. In addition to the soluble mediators, several studies have suggested that tumor-derived EVs can influence a multitude of processes that aid in tumor progression. This can be mediated by transferring bioactive cargos to recipient cells that are found in the TME [10,11,24,25]. For example, tumor-derived EVs released from the NSCLC cell line A549 were shown to affect endothelial cells and stromal fibroblasts [26]. Indeed, we previously demonstrated that EVs derived from NSCLC were internalized into MCs and activated them to release several mediators, including TNF-α and monocyte chemoattractant protein 1 (MCP-1)/ CC chemokine ligand 2 (CCL2), in addition to enhancing their chemotactic and chemokinetic activity [12].

Thus, in addition to soluble mediators in the TME, tumor-derived EVs may provide a new means to activate MCs, which in turn can affect the malignant process. However, the contribution of these potential mechanisms to the TMV-mediated activation of MCs has yet to be defined, and is the focus of our current research.

In the present study, we found additional chemokines, such as CCL4 and CCL18, that were released by MCs activated by NSCLC-derived TMVs (Figure 1).

CCL18 is expressed in various malignancies, including lung, ovarian, breast and oral squamous cell cancers, and promotes malignant behaviors in various tumors [27,28,29,30]. MCs were found to accumulate in the tumor stroma of different human cancer types in which CCL18 was highly expressed, such as lung, breast, ovarian and others [4]. CCL18 is predominantly secreted by M2 tumor-associated macrophages but is also expressed in other immune cells, such as monocytes and dendritic cells [29,31]. In NSCLC tissues, a strong expression of CCL18 is correlated with lymph node metastasis, distant metastasis and poor prognosis [13,27]. Recently, it was demonstrated that CCL18 released from tumor-associated macrophages can promote angiogenesis in breast cancer independently of VEGF-R signaling [21]. This is consistent with the results presented in the current study demonstrating that CCL18 released by NSCLC-TMV-activated MCs promoted endothelial cell migration and angiogenesis, as shown in Figure 2 and Figure 3.

In addition to CCL18, it was previously shown that the activation of bone-marrow-derived MCs by A549-derived exosomes results in MC degranulation and release of tryptase, which can promote the angiogenesis of HUVECs through the JAK-STAT signaling pathway [32]. Of note, MC activation by A549-TMVs does not induce MC degranulation [12]. This probably reflects the differences between MC origin and the type of EV used.

Surprisingly, CCL18 released by NSCLC-TMV-activated MCs did not affect endothelial cell proliferation (Figure 2C). This phenomenon was also documented by others and may be due to the initiation of differentiation processes [21]. The EMT is an essential mechanism in embryonic development and tissue repair, and contributes to the progression of diseases, including cancer [23,33]. In vascular endothelial cells, a similar transition also occurs as a result of the induction of transcription factors that alter gene expression to promote loss of cell–cell adhesion, leading to a shift in cytoskeleton dynamics and a change from epithelial morphology and physiology to the mesenchymal phenotype [34]. The EndMT has a critical role during tumor angiogenesis [35]. For example, in angiogenesis, EndMT enables tip cells to promote the appearance of the vascular plexus and migration into adjacent tissue [36,37]. Previous studies have demonstrated that CCL18 released by tumor-associated macrophages induced EndMT in endothelial cells, altering HUVEC morphology, suppressing VE-cadherin expression and increasing the levels of vimentin and fibronectin. It was also demonstrated that the expression of Snail, a transcriptional repressor associated with EndMT, was upregulated by CCL18 via activation of ERK and AKT/GSK-3β signaling pathway [21]. Here, we also show that incubating endothelial cells with supernatants obtained from NSCLC-TMV-activated MCs resulted in increased EndMT, which was determined by increased N-cadherin expression. Addition of neutralizing anti-CCL18 mAb resulted in the inhibition of N-cadherin expression, indicating the involvement of CCL18 in this process.

## 5. Conclusions

MCs can be potent inducers of angiogenesis due to their ability to synthesize and release several angiogenic factors, such as FGF, IL-8, VEGF, TGF-β, TNF-α and tryptase [6]. The results of the present study suggest that NSCLC cells release microvesicles that activate MCs to release several chemokines, among them CCL18, which thereafter serves as an angiogenic factor due to its ability to promote tumor angiogenesis. Thus, our data strengthen the conclusion that TMVs have the potential to influence MC activity and thereby affect angiogenesis in the TME.

## Figures and Tables

**Table 1:** Gene ontology (GO) analyses of the genes regulated in response to the activation of LAD2 cells by A549-TMVs.

	ToppGene	*p*-Value GOrilla	String
***Up-regulated genes*** **Cellular response to stimulus**			
Cellular response to stimulus	2.19 × 10^−6^	1.54 × 10^−6^	
Chemokine-mediated signaling pathway	7.89 × 10^−5^	1.22 × 10^−4^	
Cellular response to organic substance		1.32 × 10^−4^	
Cytokine–cytokine receptor interaction	4.59 × 10^−5^		0.0076
Interleukin-10 signaling	2.38 × 10^−4^		
Inflammation mediated by chemokine and cytokine signaling pathway	1.22 × 10^−3^		
NF-kappa B signaling pathway	1.66 × 10^−3^		0.00024
TNF signaling pathway			0.0017
IL-17 signaling pathway			0.0126
Toll-like receptor signaling pathway	1.22 × 10^−4^		
**Cell chemotaxis**		5.60 × 10^−4^	
Lymphocyte migration		5.00 × 10^−4^	
Monocyte chemotaxis		5.00 × 10^−4^	
Lymphocyte migration chemotaxis		5.00 × 10^−4^	
**Positive regulation of ERK1 and ERK2 cascade**	5.42 × 10^−5^	5.00 × 10^−4^	
**Apoptosis**	9.77 × 10^−3^		0.0119
***Down-regulated genes***			
**Cell cycle**			
Cell cycle, mitotic	7.54 × 10^−107^	1.73 × 10^−5^	3.70 × 10^−28^
Cell cycle checkpoints	9.19 × 10^−93^	3.27 × 10^−6^	
**MicroRNAs in cancer**	6.72 × 10^−26^		8.59 × 10^−5^
**DNA replication**	3.85 × 10^−27^		2.27 × 10^−16^
**p53 signaling pathway**			1.40 × 10^−5^
**Cell division**	1.41 × 10^−9^	6.65 × 10^−4^	

Differentially expressed gene cutoff: fold change, >1.5; *p* < 0.05.

**Table 2:** List of differentially expressed genes (*p* < 0.05 and fold-change 1.5) that belong to the cluster of cellular response stimulus.

*Gene Symbol*	*Description*	*Fold Change*	*p-Value*
*EGR1*	Early growth response 1	4.74	4.72 × 10^−23^
*EGR3*	Early growth response 3	3.67	9.11 × 10^−16^
*CCL4*	CC chemokine ligand 4	2.82	1.80 × 10^−10^
*CCL18*	CC chemokine ligand 18	2.59	2.72 × 10^−20^
*FOSB*	FosB proto-oncogene, AP-1 transcription factor subunit	2.53	1.13 × 10^−8^
*ATF4*	Activating transcription factor 4	2.52	1.35 × 10^−86^
*CCL4L2*	C-C motif chemokine ligand 4 like 2	2.51	8.19 × 10^−9^
*CCL3L1*	C-C motif chemokine ligand 3 like 1	2.43	3.20 × 10^−8^
*BMP7*	Bone morphogenetic protein 7	2.01	9.57 × 10^−9^
*CCL3*	C-C motif chemokine ligand 3	2.0	4.79 × 10^−6^
*TNFRSF12A*	TNF receptor superfamily member 12A	1.93	2.18 × 10^−6^
*PTGS2*	Prostaglandin-endoperoxide synthase 2	1.92	5.47 × 10^−5^
*TNF*	Tumor necrosis factor	1.89	6.20 × 10^−6^

## Supplementary Materials

The following are available online at https://www.mdpi.com/article/10.3390/cells11030353/s1. Table S1: List of upregulated genes in response to activation of LAD2 cells by A549-TMVs. Table S2: List of downregulated genes in response to activation of LAD2 cells by A549-TMVs.

## Abbreviations

CCL2	CC chemokine ligand 2
CCL4	CC chemokine ligand 4
CCL18	CC chemokine ligand 18
EndMT	Endothelial-to-mesenchymal transition
EV	Extracellular vesicle
HUVEC	Human umbilical cord endothelial cell
MC	Mast cell
MCP-1	Monocyte chemoattractant protein 1
NSCLC	Non-small-cell lung cancer
TAM	Tumor-associated macrophage
TMV	Tumor-derived microvesicle
TME	Tumor microenvironment
