# Supplementary material for: Tumor-Derived Extracellular Vesicles Induce CCL18 Production by Mast Cells: A Possible Link to Angiogenesis
Source: Cells. 2022 Jan 21;11(3):353. doi: 10.3390/cells11030353 (PMC8834361; doi:10.3390/cells11030353)
Supplement: Supplementary file 1 [file cells-11-00353-s001.zip › cells-1549751 supplementary/Table S1-supplementary.pdf]

Table S1: List of upregulated genes in response to activation of LAD2 cells by A549-TMV (fold change, >1.5; P < 0.05.)

Gene name	Fold change	P-value
**WARS**	4.81682	1.20E-123
**EGR1**	4.747762	4.72E-23
**EGR3**	3.672329	9.11E-16
**NR4A3**	3.237475	1.34E-13
**KLHDC7B**	3.140831	9.71E-13
**SLC7A11**	2.844831	4.89E-11
**CCL4**	2.829609	1.80E-10
**AC018413.1**	2.679487	1.42E-09
**CCL18**	2.596566	2.72E-20
**TRIB3**	2.574964	9.31E-15
**SNHG7**	2.55128	1.06E-49
**FOSB**	2.534071	1.13E-08
**ATF4**	2.528172	1.35E-86
**CCL4L2**	2.519323	8.19E-09
**SELENOM**	2.489205	2.97E-14
**EPB41L4A-AS1**	2.458576	2.67E-22
**MAP1LC3A**	2.447241	2.76E-12
**LINC02365**	2.44588	3.59E-16
**ZNF581**	2.435573	3.20E-16
**CCL3L1**	2.433458	3.20E-08
**PCDHB15**	2.395269	2.31E-08
**DDIT3**	2.369201	3.48E-16
**EGR2**	2.356106	5.40E-08
**HIST3H2A**	2.344537	6.51E-31
**CFAP70**	2.328838	2.07E-07
**GARS**	2.327175	2.56E-42
**RGS20**	2.323101	1.50E-07
**CCPG1**	2.284361	6.68E-17
**BIRC3**	2.258823	9.97E-11
**ATF3**	2.225794	4.19E-07
**C6orf48**	2.219108	1.12E-42
**FCRLA**	2.210092	3.09E-07
**PSAT1**	2.18568	1.42E-06
**C5orf47**	2.183919	1.30E-12
**DAAM1**	2.180419	3.59E-09
**SLC3A2**	2.175255	4.18E-18
**LINC01873**	2.168777	9.62E-12
**AKAP5**	2.168704	1.64E-08
**CADM1**	2.130143	9.89E-13
**TMEM45A**	2.113973	3.19E-07
**BEX2**	2.099039	3.14E-31
**AC087473.1**	2.096212	4.26E-13
**AC131649.2**	2.084935	2.68E-07
**RASEF**	2.079836	1.01E-06

**XPOT**	2.074602	5.73E-14
**CCDC18-AS1**	2.071087	9.08E-14
**PLIN2**	2.051607	4.49E-28
**RSL24D1**	2.043412	8.91E-29
**BMP7**	2.012223	9.57E-09
**AC006548.3**	2.006746	2.50E-10
**CCL3**	2.00189	4.79E-06
**PRNP**	2.001033	4.99E-06
**IER3**	1.992283	5.24E-09
**PCK2**	1.985291	4.53E-07
**YPEL4**	1.982208	2.57E-05
**GAS5**	1.979888	2.04E-27
**CTSF**	1.97523	3.57E-10
**EVI2A**	1.972849	5.87E-23
**CEBPB**	1.971092	1.02E-21
**MTHFD2**	1.954214	1.12E-28
**LRRC75A**	1.953826	1.39E-07
**CA5A**	1.936203	3.53E-11
**SPACA3**	1.933486	3.54E-05
**TNFRSF12A**	1.932322	2.18E-06
**RASGEF1B**	1.926406	7.75E-06
**PTGS2**	1.919871	5.47E-05
**AC093627.4**	1.919831	5.82E-05
**UBXN1**	1.913741	1.32E-27
**SNHG8**	1.913723	1.02E-18
**LY96**	1.912752	4.87E-05
**LRRC75A-AS1**	1.899769	5.17E-12
**TNF**	1.899733	6.20E-06
**SPP1**	1.895489	2.12E-05
**SARS**	1.894817	1.22E-32
**BFSP1**	1.885954	9.34E-05
**CRISPLD1**	1.88539	6.24E-05
**AC021242.3**	1.879448	0.000107096
**AC068448.1**	1.876629	5.81E-05
**CSNK2A2**	1.872943	6.55E-18
**GPR34**	1.867772	4.82E-13
**DUSP4**	1.865189	2.28E-19
**Z99755.3**	1.864255	6.75E-13
**HS3ST2**	1.862199	7.77E-12
**AC099550.1**	1.860596	1.03E-05
**AC005632.4**	1.857766	1.05E-06
**AC074349.1**	1.8557	3.30E-10
**XIRP1**	1.851535	7.82E-06
**SPRY2**	1.833385	0.000179215
**LINC00506**	1.828317	0.000187719
**PIGN**	1.827877	1.37E-07
**MIPOL1**	1.821275	6.35E-07
**DPH5**	1.8166	1.92E-15

**GZMB**	1.816203	6.91E-06
**KCNQ1OT1**	1.810482	2.81E-07
**FOS**	1.807978	9.63E-05
**TNFRSF4**	1.80637	0.000110389
**SLC7A1**	1.799737	1.37E-07
**LINC00504**	1.793325	6.53E-29
**UGDH-AS1**	1.793181	0.000163715
**BIRC7**	1.792677	8.82E-12
**TCTEX1D2**	1.791659	0.00033216
**IRS2**	1.79098	3.01E-07
**IGBP1**	1.790358	1.35E-20
**PHLDA1**	1.784328	0.000380915
**BBC3**	1.784262	1.50E-11
**C4orf32**	1.782558	1.49E-05
**ZFAS1**	1.78109	4.29E-13
**TBC1D8B**	1.779953	1.93E-07
**AC019322.4**	1.779861	3.62E-06
**AL356056.1**	1.776183	1.09E-06
**EIF1**	1.775985	2.59E-30
**GBP2**	1.773753	1.47E-05
**IL18**	1.756892	7.87E-21
**KLHL24**	1.747089	2.89E-09
**AC007333.2**	1.745276	0.00022248
**HMP19**	1.74261	0.000534821
**AL954642.1**	1.741974	0.000614701
**MARS**	1.741904	1.28E-17
**AC093525.6**	1.739609	0.000461334
**AC025048.2**	1.739124	0.000376408
**IARS**	1.737063	3.13E-09
**SORBS3**	1.735059	8.25E-07
**AC044836.1**	1.727091	0.000789641
**AC025741.1**	1.725808	0.000734384
**LYRM9**	1.724622	0.000447327
**AC091825.1**	1.722677	0.000789987
**AC108134.3**	1.722638	2.78E-09
**GPR146**	1.722554	0.000805336
**MTURN**	1.715784	7.63E-08
**AC091544.5**	1.713536	0.000658952
**TDRD3**	1.711926	1.20E-27
**SEMA6A**	1.711802	0.000540738
**GABARAPL1**	1.703206	0.000338287
**LINC00173**	1.701922	5.95E-05
**ZNF528-AS1**	1.7004	0.000388777
**RUNDC3A-AS1**	1.698274	0.000208772
**AC026904.2**	1.697644	1.61E-05
**HCG20**	1.693653	0.0007683
**AC245140.2**	1.693512	0.001221671
**RBM43**	1.693018	6.79E-05

**HOMER3**	1.692832	0.000171049
**CAPN10-AS1**	1.692357	0.001120272
**NXPH4**	1.690915	0.000567614
**TRIB1**	1.687507	0.00110435
**MIATNB**	1.686542	0.001329504
**SPP2**	1.681657	0.00137178
**CNIH3**	1.680122	4.63E-05
**PCDHB9**	1.678873	0.000702661
**SAT2**	1.674606	1.89E-07
**AL139246.6**	1.672904	0.000537993
**ZNF540**	1.672388	4.12E-06
**BSN**	1.672063	0.001584279
**FFAR1**	1.668103	0.001113593
**LINC00920**	1.666199	0.000908172
**NBR2**	1.660286	7.60E-05
**ITGBL1**	1.660027	2.65E-09
**HERPUD1**	1.657013	2.42E-20
**SAT1**	1.656564	8.04E-31
**APOC1**	1.655728	0.000238485
**GATA3**	1.654542	0.001675138
**LINC01146**	1.653358	0.001525844
**ROR1-AS1**	1.649819	0.001237154
**STAMBPL1**	1.649736	0.000427919
**ADM2**	1.647096	0.00111239
**TAF1D**	1.645023	8.32E-12
**CREM**	1.644161	0.002294443
**NR2F2-AS1**	1.642349	0.001971267
**EIF3G**	1.642036	1.78E-11
**AL117378.1**	1.641118	0.001836444
**AC004988.1**	1.638693	8.50E-09
**GDF15**	1.638668	1.15E-25
**AP000432.2**	1.638402	0.000132306
**AC144831.1**	1.63633	0.001936612
**AC016831.5**	1.635728	0.00240484
**AL118516.1**	1.634437	0.000137551
**NINJ1**	1.6311	0.000184087
**PPY**	1.629709	1.38E-07
**LRRK1**	1.629124	0.001167979
**AL355377.2**	1.628531	5.28E-06
**XBP1**	1.628117	4.37E-06
**LY6G5C**	1.624458	0.001316233
**EIF3E**	1.624277	3.97E-25
**RAB39B**	1.623393	0.000360317
**FOXO1**	1.622876	0.001479682
**EMP2**	1.621872	0.002547126
**NTNG2**	1.621755	0.002241194
**ZNF277**	1.619849	2.30E-05
**CTTN**	1.619746	0.00242586

**AP001160.4**	1.617124	0.002037834
**FYN**	1.615315	0.002992844
**NFKBIL1**	1.61283	0.000446492
**LGALS3**	1.612285	1.54E-18
**LINC01967**	1.612237	0.003219437
**CASQ1**	1.612148	2.42E-07
**SHMT2**	1.610779	1.97E-09
**EIF4EBP1**	1.60882	2.65E-11
**ENTHD1**	1.608042	2.49E-17
**NFKBID**	1.607658	0.000174618
**CCDC122**	1.607364	0.003500834
**CD69**	1.606962	7.68E-05
**SDAD1P1**	1.606667	0.003509511
**TMEM212**	1.601863	0.000502023
**FAR2P4**	1.600332	6.96E-05
**NEGR1**	1.598378	0.003679822
**CDKN1B**	1.598329	9.69E-05
**KIAA1217**	1.594955	0.000826939
**AC107958.2**	1.594209	0.004163467
**MAFF**	1.591601	0.002132146
**ZNF442**	1.589255	0.004025004
**BCAS4**	1.589208	0.003542943
**Z95114.4**	1.589135	0.004468202
**TUBE1**	1.58905	0.000866045
**ZNF667-AS1**	1.588773	2.01E-06
**TXLNB**	1.588616	0.002198823
**RPS4Y1**	1.586131	3.90E-15
**PITPNA-AS1**	1.585739	0.000871793
**RAB2B**	1.583105	3.74E-13
**HSD17B11**	1.582851	2.40E-16
**RALGPS2**	1.581455	3.71E-05
**AC074183.2**	1.581425	0.003676371
**EIF2S3**	1.578331	1.60E-09
**FABP5**	1.576252	0.004156498
**SNX18**	1.575794	0.002698382
**ANK2**	1.57554	0.002866006
**LINC01918**	1.575508	1.75E-08
**TFPI**	1.574598	0.003929292
**HELLPAR**	1.573158	3.25E-07
**CREB3L3**	1.573129	0.000595719
**CPB2-AS1**	1.572856	0.004504779
**PIM3**	1.572574	4.58E-07
**ZFAND1**	1.57226	1.14E-05
**UBXN11**	1.571754	8.47E-05
**LINC00520**	1.570828	2.00E-11
**ANKRD42**	1.570133	0.000353509
**AC147067.1**	1.569705	0.000586265
**AC027279.1**	1.568423	0.00387749

**SPTLC3**	1.5679	0.005251628
**VIM**	1.566391	3.45E-13
**BCL11A**	1.566225	0.005878811
**AC092119.2**	1.565452	0.004154722
**S100Z**	1.564192	0.000229983
**UCKL1-AS1**	1.563222	0.006101389
**APOL2**	1.562939	0.003376997
**AC121333.1**	1.562688	0.006000649
**TIMM9**	1.5626	2.95E-05
**TMEM182**	1.561024	0.004093834
**NEDD4**	1.560643	0.004686409
**TXNIP**	1.559466	7.64E-12
**H1F0**	1.558846	9.15E-14
**LINC01481**	1.557685	0.001863174
**AC015883.1**	1.555972	0.006628914
**NPY2R**	1.55567	0.000361133
**CYB561D2**	1.555436	0.006467591
**SNHG19**	1.554631	0.000193573
**C11orf1**	1.554267	1.11E-08
**AZU1**	1.553899	0.001065636
**NDFIP2**	1.552935	7.10E-05
**LINC01023**	1.551481	0.000219413
**BCAS1**	1.551131	0.006988641
**ATXN3**	1.550259	1.30E-08
**SRP14-AS1**	1.548291	0.004376626
**AC083862.2**	1.548111	0.005264277
**ISCU**	1.547278	1.73E-12
**DALRD3**	1.545288	1.16E-06
**AL591441.1**	1.544613	0.006118683
**NCOA7**	1.544155	1.91E-05
**OSBPL6**	1.542868	0.005575635
**AL022311.1**	1.542831	0.000224502
**KRBOX4**	1.541595	0.001276785
**AC104662.3**	1.541308	0.000612139
**CALCOCO1**	1.541255	3.63E-07
**SNHG12**	1.540664	2.63E-05
**NEAT1**	1.540653	2.15E-07
**HIST2H2BE**	1.539978	0.001630416
**NAB2**	1.539752	0.000569845
**NATD1**	1.539553	0.007921278
**JMY**	1.53916	0.008135075
**RPL5**	1.537645	1.71E-20
**AC118344.2**	1.537632	0.003058323
**MPV17L**	1.536985	1.00E-05
**CSMD1**	1.536782	0.005588594
**NR4A2**	1.535989	0.005283919
**LETMD1**	1.535525	1.06E-05
**AC008074.3**	1.533648	0.008053532

**TPT1-AS1**	1.533015	0.000865021
**SSC5D**	1.532679	0.005337686
**CD22**	1.532189	7.04E-14
**DNM1P35**	1.531087	0.001362189
**LOH12CR2**	1.531029	0.005123265
**ACSL1**	1.530405	0.000503658
**SRGAP1**	1.529715	0.000105406
**AC008079.2**	1.52887	0.005841635
**AC020916.1**	1.528059	0.006407453
**CREBRF**	1.526617	1.51E-09
**FMNL2**	1.525467	0.006351447
**SLC2A11**	1.524341	1.31E-06
**Z95114.1**	1.524236	0.002814622
**P3H1**	1.522736	0.004871039
**ELP2**	1.522164	0.000445534
**ZNF471**	1.522128	0.003804882
**ZNF331**	1.521475	0.001236185
**LINC00900**	1.521474	0.000644537
**NPM1**	1.521333	2.33E-12
**LINC00694**	1.519657	0.002761386
**LINC00672**	1.518487	0.00816387
**RAB11FIP1**	1.518403	4.04E-05
**EN2**	1.518287	0.007421717
**YARS**	1.51774	7.55E-09
**RILPL1**	1.516226	0.000140891
**HIC1**	1.515921	0.006119431
**GIN1**	1.515286	0.003865018
**ZNF677**	1.515112	0.006024959
**DNAH1**	1.515104	0.005538778
**IL1B**	1.513992	0.00104329
**KLF10**	1.513889	0.001435195
**AC092068.3**	1.513473	0.007737754
**CD55**	1.512531	1.78E-07
**TM4SF19-AS1**	1.512257	0.007352596
**LINC02099**	1.510545	2.40E-05
**CHMP1B**	1.51026	5.51E-06
**ERP29**	1.509261	8.95E-11
**RPS6KL1**	1.509132	0.006115793
**AC022400.7**	1.508968	4.16E-06
**PIGP**	1.508635	0.000374513
**COL28A1**	1.505352	0.008463562
**XIAP**	1.505122	5.22E-09
**AL672032.1**	1.501879	0.006551822
**ARHGAP12**	1.501098	0.00812918
**SESN2**	1.500479	0.000117007
