# Supplementary material for: Tumor-Derived Extracellular Vesicles Induce CCL18 Production by Mast Cells: A Possible Link to Angiogenesis
Source: Cells. 2022 Jan 21;11(3):353. doi: 10.3390/cells11030353 (PMC8834361; doi:10.3390/cells11030353)
Supplement: Supplementary file 1 [file cells-11-00353-s001.zip › cells-1549751 supplementary/Table S2-supplementary.pdf]

Table S2: List of downregulated genes in response to activation of LAD2 cells by A549-TMV (fold change, >-2; P < 0.05.)

Gene name	Fold change	P-value
**TK1**	-8.45245	1.10E-87
**MKI67**	-8.24437	1.89E-113
**TOP2A**	-7.29947	2.13E-120
**RRM2**	-7.2316	3.28E-58
**TYMS**	-6.374	7.67E-112
**PRC1**	-6.25551	9.18E-83
**TROAP**	-6.07192	6.58E-52
**BUB1B**	-6.05145	3.13E-53
**PCLAF**	-6.04503	3.23E-82
**DHFR**	-6.00633	7.24E-88
**ASF1B**	-5.97451	1.71E-116
**ZWINT**	-5.95396	7.31E-50
**CDK1**	-5.81778	6.27E-63
**NUSAP1**	-5.7288	3.31E-58
**DERL3**	-5.68691	3.16E-44
**GTSE1**	-5.58355	1.57E-34
**SKA1**	-5.3577	3.31E-43
**KIFC1**	-5.31479	3.48E-47
**UBE2C**	-5.31132	2.56E-69
**NCAPG2**	-5.30366	1.94E-35
**ESPL1**	-5.18793	6.57E-30
**CENPF**	-5.18401	1.65E-58
**CENPM**	-5.1498	2.43E-37
**CDCA5**	-5.14721	8.47E-29
**ZNF367**	-5.1122	2.16E-30
**CCNA2**	-5.10357	1.19E-56
**E2F1**	-5.0998	1.49E-43
**TMEM97**	-5.04279	1.48E-81
**CEP55**	-5.01077	6.94E-36
**CDKN3**	-5.00966	1.36E-40
**TPX2**	-5.00231	1.16E-35
**HMMR**	-4.99963	4.77E-41
**BUB1**	-4.89036	8.83E-31
**FEN1**	-4.83472	4.52E-72
**NDC80**	-4.79878	3.31E-31
**RAD51AP1**	-4.79167	1.60E-28
**PBK**	-4.75663	1.03E-30
**ESCO2**	-4.75359	6.27E-31
**CCNB1**	-4.72447	6.37E-52
**MELK**	-4.69593	1.10E-26
**AURKA**	-4.6321	8.94E-37
**PIMREG**	-4.62143	3.06E-27
**ASPM**	-4.57793	5.10E-32
**PKMYT1**	-4.56919	3.37E-40

**SPC25**	-4.51849	8.90E-32
**CDCA3**	-4.51377	3.82E-24
**KIF23**	-4.44673	2.04E-25
**CDC6**	-4.41259	5.94E-25
**GGH**	-4.39656	2.78E-62
**CDCA8**	-4.38675	5.13E-32
**NUF2**	-4.36007	1.25E-35
**NCAPG**	-4.33852	8.10E-27
**PRR11**	-4.30153	1.84E-48
**MCM5**	-4.26704	7.85E-51
**HELLS**	-4.22062	2.47E-32
**CENPK**	-4.21386	1.04E-42
**RFC3**	-4.20748	3.01E-44
**CENPW**	-4.08753	8.20E-26
**SHCBP1**	-4.07597	6.62E-39
**MCM7**	-4.03061	1.05E-85
**BIRC5**	-3.92396	6.04E-26
**GINS2**	-3.89752	1.04E-27
**TCF19**	-3.88305	2.05E-21
**KIF4A**	-3.86639	8.80E-22
**AL109918.1**	-3.86337	4.63E-18
**FANCA**	-3.85518	5.12E-19
**KNL1**	-3.84755	2.35E-20
**KIF18A**	-3.83272	3.24E-20
**PLK4**	-3.79173	2.17E-20
**FBXO5**	-3.78291	5.33E-24
**OIP5**	-3.7804	1.05E-18
**RAD51**	-3.77255	2.12E-21
**MAD2L1**	-3.74654	1.74E-34
**CDC45**	-3.71273	2.30E-18
**DSCC1**	-3.71156	4.04E-19
**ATAD2**	-3.70434	2.55E-28
**ARHGAP11A**	-3.70117	2.81E-22
**TUBA1B**	-3.69597	1.40E-93
**CDC25A**	-3.68154	7.73E-17
**CCNB2**	-3.67444	3.44E-29
**MCM10**	-3.65744	1.26E-18
**HJURP**	-3.62821	5.49E-21
**CIT**	-3.62389	1.88E-18
**MYBL2**	-3.61864	6.82E-17
**ANLN**	-3.60746	7.04E-16
**CENPA**	-3.60496	4.12E-18
**SGO1**	-3.59817	2.65E-21
**CHEK1**	-3.58823	1.85E-25
**TRIP13**	-3.56361	1.60E-23
**MCM4**	-3.54755	1.80E-26
**FANCI**	-3.53919	2.06E-16
**PLK1**	-3.53361	1.30E-40

**CCDC34**	-3.51217	5.84E-17
**SKA3**	-3.50064	2.23E-16
**CKAP2L**	-3.4874	5.49E-26
**CHAF1A**	-3.4716	2.10E-21
**HIST1H4C**	-3.4464	1.11E-22
**CKAP2**	-3.41391	3.34E-33
**UHRF1**	-3.401	2.77E-14
**CCNE2**	-3.37131	1.57E-18
**FASN**	-3.36699	2.77E-30
**PTTG1**	-3.35002	2.34E-40
**KIF2C**	-3.32026	1.43E-16
**SPC24**	-3.30877	3.76E-14
**ACAT2**	-3.30548	1.08E-73
**RACGAP1**	-3.29303	1.46E-24
**FOXM1**	-3.28701	6.33E-17
**NCAPH**	-3.28433	2.28E-15
**RAD54B**	-3.25717	4.00E-14
**H2AFX**	-3.23173	4.10E-31
**KIF15**	-3.21799	6.84E-13
**MCM8**	-3.2168	3.26E-23
**GINS1**	-3.20839	1.19E-16
**ORC6**	-3.2024	2.39E-30
**CENPU**	-3.19185	4.22E-19
**SPAG5**	-3.17759	3.25E-15
**MCM2**	-3.16948	4.48E-36
**TACC3**	-3.16197	1.47E-26
**SMC4**	-3.14112	9.68E-41
**STMN1**	-3.13129	1.56E-54
**CKS1B**	-3.11731	4.18E-46
**CENPE**	-3.0993	6.67E-15
**KIF11**	-3.0952	4.65E-19
**CDK2**	-3.09292	1.24E-15
**RECQL4**	-3.09079	2.24E-14
**PSMC3IP**	-3.0876	1.64E-13
**FDPS**	-3.0824	7.38E-79
**CDKN2C**	-3.02892	6.20E-32
**ATAD5**	-3.02513	2.86E-13
**NEURL1B**	-3.01682	1.23E-11
**CLSPN**	-2.99189	4.40E-16
**WDR76**	-2.98172	2.69E-12
**DDIAS**	-2.96239	4.72E-12
**SAPCD2**	-2.95908	1.97E-15
**UNG**	-2.95511	2.32E-21
**BRCA1**	-2.94454	2.28E-24
**PCNA**	-2.9327	5.78E-35
**RFC4**	-2.93121	2.25E-22
**UBE2T**	-2.91879	1.56E-20
**MXD3**	-2.88952	2.88E-14

**POLD1**	-2.88928	6.24E-13
**MCM3**	-2.88371	1.75E-30
**E2F2**	-2.86331	2.10E-11
**HMGB2**	-2.85602	5.55E-29
**LIG1**	-2.84094	4.15E-17
**TTK**	-2.83784	2.64E-14
**TMEM106C**	-2.81356	1.71E-24
**EZH2**	-2.81279	5.65E-19
**KIF14**	-2.80914	3.88E-14
**C21orf58**	-2.79132	1.10E-11
**POLE2**	-2.78943	1.21E-10
**KIAA1524**	-2.78779	1.18E-10
**SAC3D1**	-2.76446	2.62E-21
**DDX11**	-2.75253	4.07E-16
**FAM111B**	-2.75069	3.68E-10
**CDCA2**	-2.70565	9.63E-10
**DSN1**	-2.7011	8.16E-14
**SPDL1**	-2.69819	7.62E-12
**C4orf46**	-2.69458	1.57E-11
**RMI2**	-2.69357	3.91E-14
**HMGCS1**	-2.6912	1.85E-81
**NUDT8**	-2.68997	2.51E-11
**PSRC1**	-2.68935	2.77E-10
**MASTL**	-2.66703	6.02E-11
**NEK2**	-2.64429	1.69E-11
**NCAPD2**	-2.63608	1.31E-19
**RRM1**	-2.62975	7.71E-24
**FAM83D**	-2.6203	2.23E-09
**DTL**	-2.61082	3.81E-10
**LSS**	-2.59763	7.01E-22
**WEE1**	-2.58537	9.84E-16
**STIL**	-2.57806	8.39E-12
**MIS18A**	-2.56308	5.65E-13
**DIAPH3**	-2.55395	5.32E-09
**TUBB4B**	-2.53006	1.54E-50
**KIF20B**	-2.51961	4.57E-14
**TUBB**	-2.5105	2.41E-37
**BCL2L12**	-2.50658	5.56E-21
**DNMT1**	-2.49922	1.60E-23
**RAD54L**	-2.47935	1.88E-08
**NRM**	-2.47716	3.35E-13
**TM7SF2**	-2.47646	1.03E-14
**SMC2**	-2.47017	2.41E-23
**CDT1**	-2.46659	3.61E-13
**AURKB**	-2.45746	8.47E-12
**POLA2**	-2.45665	5.52E-10
**POLA1**	-2.45561	3.18E-09
**TUBA1C**	-2.43857	4.18E-44

**TEX30**	-2.42791	4.02E-14
**POLQ**	-2.42146	1.59E-08
**CCNF**	-2.42034	9.38E-10
**DLGAP5**	-2.41868	6.04E-08
**CTPS1**	-2.40706	4.96E-08
**FIGNL1**	-2.40683	2.34E-09
**EXO1**	-2.40488	7.36E-08
**DTYMK**	-2.40089	2.33E-15
**CDCA7**	-2.399	1.58E-11
**NET1**	-2.39686	2.81E-09
**MND1**	-2.39274	4.03E-08
**BLM**	-2.36339	6.09E-10
**NUDT15**	-2.36041	4.86E-11
**PHF19**	-2.3514	8.87E-11
**CENPO**	-2.34902	5.90E-08
**CSE1L**	-2.34014	4.24E-18
**NDC1**	-2.32819	6.40E-13
**AACS**	-2.32336	5.01E-16
**MCM6**	-2.31722	3.26E-11
**CDC20**	-2.30541	1.79E-08
**SLF1**	-2.29843	3.09E-09
**TIMELESS**	-2.29333	1.27E-14
**KIF18B**	-2.28394	3.95E-07
**KNSTRN**	-2.28337	4.98E-12
**DHCR7**	-2.28335	2.24E-22
**RMI1**	-2.26531	7.12E-25
**EBP**	-2.25558	1.45E-46
**RBL1**	-2.24857	6.50E-09
**RDM1**	-2.24086	6.35E-07
**GMNN**	-2.23602	1.68E-12
**C16orf59**	-2.22633	8.35E-07
**EMC3-AS1**	-2.22358	3.54E-07
**TUBG1**	-2.2183	4.98E-14
**CARHSP1**	-2.21738	9.53E-13
**MVK**	-2.20784	1.14E-10
**NCAPD3**	-2.20685	3.61E-12
**CHTF18**	-2.20653	1.14E-06
**ERCC6L**	-2.18754	1.57E-06
**ELOVL6**	-2.18449	2.27E-18
**E2F8**	-2.17816	1.59E-06
**C14orf1**	-2.17802	5.47E-40
**CEP152**	-2.17767	1.55E-08
**DEPDC1B**	-2.17401	5.99E-12
**FADS2**	-2.17099	8.23E-28
**FANCG**	-2.17023	9.03E-15
**SNRNP25**	-2.16775	9.30E-15
**GINS3**	-2.1669	1.02E-09
**ZWILCH**	-2.16388	7.77E-12

**SASS6**	-2.16294	4.60E-07
**HMGCR**	-2.16201	1.16E-25
**CDKN2A**	-2.15701	9.02E-19
**AL035461.2**	-2.15104	2.51E-06
**BARD1**	-2.14603	2.43E-09
**LMNB2**	-2.14542	4.24E-07
**CENPN**	-2.13333	3.62E-07
**NEIL3**	-2.1233	3.85E-06
**WDR90**	-2.12281	2.00E-07
**TMPO-AS1**	-2.11629	2.35E-06
**HES6**	-2.10966	4.53E-07
**TMPO**	-2.10956	1.57E-18
**ASRGL1**	-2.10539	9.57E-10
**DHCR24**	-2.10312	2.07E-18
**MYBL1**	-2.09434	5.76E-06
**MNS1**	-2.09208	4.02E-06
**LRP8**	-2.09071	2.18E-11
**RNASEH2A**	-2.08831	1.66E-16
**COQ2**	-2.07647	4.06E-06
**KMT5A**	-2.07163	1.18E-07
**HDGF**	-2.06255	3.56E-21
**WRAP53**	-2.06092	1.84E-06
**JSRP1**	-2.06078	8.02E-13
**TYMSOS**	-2.05978	8.98E-06
**INSIG1**	-2.05965	1.40E-36
**GEN1**	-2.05916	2.23E-08
**AC012073.1**	-2.05746	7.72E-06
**CCDC18**	-2.056	2.55E-06
**ITGB3BP**	-2.05338	3.36E-08
**FADS1**	-2.05278	2.41E-40
**ARHGAP11B**	-2.04075	2.77E-06
**THOC3**	-2.02729	7.19E-13
**LRR1**	-2.02477	1.72E-12
**ECT2**	-2.01766	1.23E-05
**RAD18**	-2.00499	3.24E-06
**CENPH**	-2.0046	3.84E-08
**C9orf40**	-2.00167	1.59E-06
